# PANDEMIC-RELATED SOCIAL RESTRICTIONS INCREASED THE URGENCY AND ENGAGEMENT IN OLD-AGE PREPARATION

**DOI:** 10.1093/geroni/igac059.504

**Published:** 2022-12-20

**Authors:** Yaeji Kim-Knauss

**Affiliations:** Friedrich-Alexander University Erlangen-Nuremberg, Nuremberg, Bayern, Germany

## Abstract

We investigated whether people who perceive more restrictions on social contacts during the pandemic set an earlier deadline to prepare for social connectedness in old age (i.e., the latest still good age to start preparation) than they had perceived in the pre-pandemic time. We also looked at whether this change in the deadline induces the engagement in the preparation concerning the same domain. We first fit the data obtained from 356 German adults in 2018 and 2020 (aged 22─95 years) to a change score model. The deadline for preparing for social connectedness in old age was set about 23 years earlier in 2020 compared to that reported in 2018. We found that perceiving more social restrictions during the pandemic predicted this shorter deadline, which in turn, induced greater engagement in the preparation. A possible consequence of the pandemic may be related to an increased motivation to prepare for old age.

